# Insights from mathematical modelling and quantitative analysis on the proposed 2030 goals for trachoma

**DOI:** 10.12688/gatesopenres.13086.2

**Published:** 2021-03-11

**Authors:** NTD Modelling Consortium discussion group on trachoma

**Keywords:** Trachoma, Elimination as a public health problem, mass drug administration, surveillance, monitoring and evaluation

## Abstract

Trachoma is a neglected tropical disease and the leading infectious cause of blindness worldwide. The current World Health Organization goal for trachoma is elimination as a public health problem, defined as reaching a prevalence of trachomatous inflammation-follicular below 5% in children (1-9 years) and a prevalence of trachomatous trichiasis in adults below 0.2%. Current targets to achieve elimination were set to 2020 but are being extended to 2030. Mathematical and statistical models suggest that 2030 is a realistic timeline for elimination as a public health problem in most trachoma endemic areas. Although the goal can be achieved, it is important to develop appropriate monitoring tools for surveillance after having achieved the elimination target to check for the possibility of resurgence. For this purpose, a standardized serological approach or the use of multiple diagnostics in complement would likely be required.

## Disclaimer

 The views and opinions expressed in this article are those of the author(s) and do not necessarily reflect the views of the World Health Organization. Publication in Gates Open Research does not imply endorsement by the Gates Foundation.

## Background

Trachoma is a neglected tropical disease caused by infection with the bacterium *Chlamydia trachomatis*. During an infection episode, conjunctival inflammation occurs, which leads to the presence of follicles on the eyelids (Trachomatous inflammation-follicular (TF)) ^1^. Repeated infection with the bacteria over time, which results in scarring of the eyelids, leads to in-turning of the eyelashes. This is known as trachomatous trichiasis (TT), which traumatizes the eye surface leading to superinfection and blindness ^1, 2^.

The World Health Organization leads an Alliance that aims to achieve the elimination of trachoma as a public health problem (EPHP) in all endemic districts by 2020 ^1^. This is defined by the achievement of three goals: 1) reduction of TF prevalence in 1–9 year olds to <5% in each formerly endemic district, 2) a TT prevalence “unknown to the health system” in >=15-year-olds of <0.2% , and 3) the presence of a system to identify and manage incident cases of TT. In order to eliminate trachoma, the WHO endorses the implementation of the SAFE strategy which consists of four components: (S) surgery to correct trichiasis; (A) mass distribution of antibiotics to clear infection in the community (topical tetracycline is used in very young children or other individuals unable to take azithromycin), (F) promotion of facial cleanliness in order to reduce transmission via eye discharge and (E) environmental improvement to ensure that the environment no longer helps to facilitate the transmission of infection. Currently, the prevalence of clinically active trachoma is assessed by trained graders’ clinical examination. In the context of low endemicity, other methods are being considered, including photographic and laboratory assessment. MDA is recommended to all districts where TF is >5%. A course of one, three, or five annual rounds is recommended where TF is between 5–10%, 10–30%, and over 30% respectively. Following completion of the prescribed MDA, a follow-up survey is conducted to assess whether further rounds of MDA are required. To date, ten countries have been validated by WHO as having achieved EPHP ^3^.

Both mathematical and statistical models have been developed to gain insight into the transmission dynamics of infection. Such models have been used to try and understand the potential impact of different intervention strategies that could help to accelerate elimination efforts ^4^, as well as understanding likely elimination timelines through forecasting. In addition, a recent review on the contribution of mathematical modelling to trachoma research and elimination efforts was published by the two teams in the first iteration of the NTD modelling consortium ^5^. Furthermore, a multi-group forecast comparison was also conducted to look at the strengths and limitations of different modelling approaches for forecasting the future prevalence of TF at the district level ^6^.

Moving forward past the current 2020 goals, whilst substantial progress has been made towards achieving EPHP of trachoma, it has become apparent that a number of endemic regions will not achieve this target by 2020. It is important to note that the original 2020 goals were political and aspirational, and thanks to the effort of ministries of health and countless donors and partners over the years can now be formally assessed with data. Therefore, WHO has revised the timeline, with the aim of validating EPHP in all endemic countries by 2030. Using the insights that have been gained from recent modelling work on trachoma, in this article we highlight the practical considerations of EPHP (the timelines required, sufficiency of current surveillance diagnostics and feasibility of achieving it) and the future considerations that may be needed following EPHP to maintain the gains ( Table 1 provides a summary of the key issues).

**Table 1.** Executive summary.

Current WHO Goal	Validation of EPHP (TF threshold <5% in children, TT prevalence unknown to the health system in adults >=15 years of age of 0.2%).
2030 Target	Validation of EPHP for all countries, including identification and management of incident TT cases.
Is the new target technically feasible under the current disease strategy?	Except in certain hyperendemic settings (>40% TF prevalence) EPHP is achievable using public health-level TT surgical services + annual MDA alone.
If not, what is required to achieve the target? (updated strategy, use of new tools, etc.)	Enhanced MDA campaigns to reduce the TF prevalence.
Are current tools able to reliably measure the target?	No. It is currently reported to be very unreliable. Standardization of grading by using smartphone photography may help to improve reliability. There are also technical challenges in measuring TT prevalence with useful precision.
What are the biggest unknowns?	1) The best strategy to monitor the disease after EPHP has been achieved. 2) If or how does F&E contribute to the achievement and maintenance of EPHP.
What are the biggest risks?	Resurgence after achieving EPHP; insufficient treatment/lack of understanding about what is happening in settings where transmission persists at a moderate level despite 10 rounds of MDA.

## What have we learned from the past 10 years that we can apply for the next 10 years?

There has been a substantial amount of programmatic success in trachoma elimination over the last 10 years, with global prevalence falling dramatically as a result of successful intervention programs. Whilst EPHP includes both TF and TT, most research focuses on changes, modelling and monitoring of TF and infection prevalence. Limited analysis and forecasting of TT prevalence to date has occurred in part because the trajectory of changes in observed TT prevalence depends not only on the incidence of TT (a chronic condition and stochastic process related to an individual’s past number of infections ^7^), but also due to demography and health service access. Thus the observed prevalent number of TT cases is partly determined by the speed and efficiency of active case finding and surgical service delivery, which are inherently more challenging and uncertain to model.

Mathematical modelling and current surveillance data suggests that EPHP is feasible, and indeed has already been achieved by a number of endemic countries ^3^. However, in health districts with long-term persistence, such as a few high prevalence districts in Ethiopia (>40% baseline prevalence), annual MDA alone is not sufficient to achieve EPHP and must be supplemented with additional tools to reduce transmission ^6, 8– 10^. In particular, more intensive facial cleanliness and environmental improvement (F&E) or more intensive antibiotics are measures that may be necessary in a select few hot spots ^4, 8, 11^. Similarly, statistical analysis of data provided and collected by trachoma endemic countries has indicated that the vast majority of endemic health districts are on track to achieve TF <5% for EPHP by 2020 ^6, 8^. These findings are consistent across both dynamic and statistical modelling frameworks that were independently developed by the different partners of our consortium.

In health districts that remain problematic, to understand how EPHP may be achieved by 2030, dynamic modelling work has explored a range of alternative and more intensive antibiotic distribution strategies that could be implemented ^4, 12^. To date it has been challenging to measure the true impact of F&E and its potential role in helping to reduce transmission, and thus it has been challenging to model. A few field studies that have assessed F&E were unable to find a significant effect ^13– 15^. However, an on-going clinical trial is seeking to help address this gap in knowledge (Stronger SAFE). Even if annual mass antibiotic treatment is insufficient to achieve EPHP goals in certain hyperendemic areas, it may prevent resurgence of infection ^16^.

Modelling has also been used to investigate whether targeting a residual core group of children with additional antibiotic treatment, while continuing annual MDA to the entire population would be more effective at clearing infection from the community ^17^. The study suggested that if average duration of infection per group and dominant eigenvalue of a next generation matrix of the transmission model are defined, then a sufficient core group can be determined and used to find the absolute minimum sized core group, based on a fully specified model or even from epidemiological data. A separate mathematical model of a double-dose antibiotic treatment strategy where two doses of antibiotics are given two weeks apart, in combination with enhanced F&E suggested that feasibility of EPHP may be increased in high transmission settings ^18^. This modelling suggested that sustained F&E could help maintain the gains initially achieved through intense antibiotic distribution ^18^.

A number of RCTs informed by modelling are currently underway in Ethiopia, with the aim of assessing the potential impact of alternative and intensive treatment strategies. One RCT (KETFO) is assessing whether quaterly treatment of children alone can lead to EPHP in severely affected communities ^19^. Additionally, an RCT looking at intensive WASH (SWIFT-WUHA) ^20^ and an RCT looking at the distribution of two doses of antibiotics one week apart (TESFA ^21^) are in progress.

## What are the practical considerations of the currently proposed goals?

### Measuring the target of EPHP using TF prevalence

The current monitoring and TF survey design has been useful to predict large-scale trends and to estimate health district level prevalence of TF ^1^. However, as TF prevalence continues to decline, fewer cases are available to train graders and the decreasing severity of cases decreases make them harder to confirm. Thus, the sensitivity and specificity of the eye examination may decline. Equally important, noise due to sampling variation increases as prevalence decreases ^22^.

Complete cost-effective modelling work is yet to be published, but using TF surveillance for the current end goal is becoming more expensive ^23^. Additionally, recent epidemiological studies in the South Pacific have highlighted that TF is apparent within communities in the absence of being able to identify *C. trachomatis* through PCR ^24^. This could be due to the fact that at the community level, TF resolves slowly ^25^. This has led the community to start considering whether evaluation by PCR or through serology may be more appropriate as prevalence continues to decline ^26, 27^. However, limited data with all three diagnostics where TF is ~5% have been available to understand how all diagnostic indicators relate to each other at low prevalence. Some recent modelling sought to evaluate the relationship between TF and serological prevalence ^28^; however, more data are needed to test the robustness of these findings. Collectively, current modelling and surveillance data suggest that as we move towards 2030, the TF prevalence target may need to vary by health district and be tailored to the underlying epidemiology of certain areas.

### Ability to sustain achievement of the goal

Trials and longitudinal studies have found that after MDA, infection can return ^9, 16, 29^ in locations where TF prevalence had not declined to <5% ^16, 29^. It has been suggested that infection could re-emerge due to the loss of age-specific immunity as transmission reduces ^30^, however to date re-emergence has not been detected in districts that have eliminated trachoma (TF < 5%), that cannot be explained by misclassification error ^31^. Since TF prevalence is a lagging indicator, TF-driven programmatic activities may continue long enough to frequently achieve near elimination of *C. trachomatis* infection.

Demonstrating that the causative agent of infection is absent in endemic or formally endemic communities is the key indicator of breaking transmission. PCR as an alternative indicator for detecting resurgent infection has a number of problems, not least the short duration of infection, which limits the time-window it can be detected ^22^. Modelling work has shown that including PCR data does not significantly improve forecasts of TF ^32^. Moreover, it can be fairly costly and requires specialized equipment and technicians. However, capacities in many trachoma-endemic countries are improving. In the absence of dedicated post-elimination TF or PCR prevalence surveys, serological studies may be able to detect resurgence in transmission despite imperfect antibody specificity ^28^.

## What risks need to be mitigated to achieve and maintain the 2030 goals?

There are a number of practical factors that may directly impact on-going program implementation. Firstly, both empirical data and dynamic modelling have suggested that in areas of high prevalence, annual MDA alone is not sufficient to reach the goal ^5, 11, 33^. As previously described, a number of alternative intervention strategies are currently being evaluated within RCTs to identify solutions to this problem. Secondly, maintaining and optimising the frequency of antibiotic use is of paramount importance in order for gains to be achieved and maintained. Coverage is often reported to be high ^34^, but in practice this can be hard to measure in the field ^35^. Equally, systematic variation within Health districts leading to local pockets of undelivered MDA and exclusion from TF surveys, may limit progress by leaving reservoir sources of infection in communities that are deemed to have been treated ^36^. Thirdly, no resistance to azithromycin has been reported, however careful monitoring for suboptimal treatment effects is needed. If resistance does emerge, EPHP success will be severely undermined ^5^. Fourthly, as prevalence begins to decline in many endemic regions, movement of individuals between infected and uninfected areas may facilitate persistence of infection or re-introduction into formerly infection-free areas.

A number of risks remain for surveillance in terms of classifying and continuing to confirm elimination. First of all, it is currently uncertain whether or not TF prevalence alone is sufficient to classify health districts that have achieved EPHP ^28, 37, 38^. Meanwhile, the non-linear relationship between viral load and TF complicates our understanding of how PCR detection and TF relate to one another. In fact, TF has been detected in some areas of the world without the bacterial organism being identified, suggesting that other factors besides trachoma may also cause TF. In short, following EPHP, it is unclear how to conduct surveillance to ensure that EPHP is maintained. Serology has been suggested as one potential option, although sero-surveillance data in EPHP settings are only starting to become available.

There are a number of risks that we need to be mindful of with respect to modelling trachoma and interpreting model outputs. It is typically assumed that the accuracy of TF detection will remain constant. However, this is an optimistic assumption, as we expect the ability to recognize TF to decrease as the disease becomes rarer. This issue will become particularly important as modelling surveillance in very low transmission scenarios receives greater attention. Importantly, there are no high-resolution empirical studies on dynamics of infection in areas with hypo-endemic disease, which means that simulations and models of low-level prevalence are likely to have a large number of uncertainties. Further empirical studies are needed in order to understand how to more accurately model transmission at low prevalence.

## Future directions

### What kind of new diagnostics could be used for post-validation surveillance?

As prevalence and transmission of trachoma declines, the specificity of TF as a diagnostic indicator of conjunctival CT infection is also reported to decline ^38, 39^. Equally, following elimination of TF there is likely to be limited funding dedicated to TF surveillance to monitor and verify elimination. Therefore, it will be important to understand what alternative diagnostics, such as serology, can tell us about the prevalence and transmission of trachoma.

If serology is informative, the opportunity for trachoma post-validation surveillance increases as dried blood spots collected for other health programs might be screened for trachoma antibodies ^40– 42^. As such, although not specifically within the 2030 targets, research into the utility of sero-surveillance for understanding and quantifying transmission is important for trachoma elimination. A number of individual modelling analyses have been conducted to try and estimate sero-conversion rates (SCRs) for trachoma within different settings ^40, 41, 43^. However, individual modelling analyses of datasets in isolation make it difficult to understand the global picture. A more recent modelling analysed TF prevalence and serology data from a number of endemic regions ^28^ to estimate the SCRs and correlate these with the reported TF prevalence. This work was the first attempt to estimate an operational threshold for serology for trachoma programs. Modelling suggested that SCRs below 0.015 (95% confidence interval (CI): 0.0–0.049) per year corresponded to a prevalence of TF below 5% ^28^. Additionally, a statistical analysis suggested that sero-surveillance would require smaller sample sizes than TF surveillance because sero-prevalence is higher than the TF prevalence ^28^.

Further work is required before serology can be recommended as a post-validation surveillance tool. One existing limitation is that current analyses are being done using bead-based multiplex immunoassay systems, ELISA and lateral flow assays; standardization would aid comparison between sites. Additionally, it is unclear exactly what the quantitative population-level serological profile is expected to be in areas with sustained EPHP. A greater understanding of this is required before one can interpret serological data for trachoma in the context of post-EPHP surveillance.

### What questions can modeling help address?

In discussion with WHO, a number of priority issues and questions for trachoma control programs were identified. These questions are summarized in Table 2 and describe how mathematical and statistical modelling can help address them.

**Table 2.** Priorities issues and how modelling can help to address them.

Priority issue/question identified in discussion with WHO	How can modelling help?
Forecasting expected timeline to reach the goals	Probabilistic forecasts can be developed using statistical and mechanism-based models. However, these forecasts must be taken with caution by understanding the assumptions made and the uncertainty in the predicted outcomes.
How likely/unlikely is resurgence, how quickly is it likely to emerge and be detected and where is it more likely to emerge?	One approach is to analyze data from districts that return to TF prevalence >5% and compare it with outputs from resurgence in stochastic models. Our group has been working on assessing the likelihood of true resurgence versus misclassification error, using data collected by Trachoma endemic countries and adapting a stochastic version of the population-based model in 22, 32. To better understand timeliness of resurgence and where it is more likely to occur, scenario-based simulations could be potentially used. To inform such a model, a review of empirical studies is required, which can help inform the spatial variability. These models would benefit from including diagnostics in an explicit manner, so that surveillance approaches and detection of resurgence can be appropriately assessed.
A geospatial survey design for TT	To produce a geospatial survey design, geostatistical models can be used that can account for both spatial and temporal uncertainty in the TT estimates. This will improve survey design and will lead to a better understanding of the needs at fine geographical scales ^44^. However, this approach requires spatially explicit data.
What is the utility of serology in Identification of current hot spots and future resurgence	Modelling work has been carried out to analyze whether serological data is informative of patterns of transmission and whether it could be used to inform programmatic decisions ^28^. More serological data will be available in the future that can be integrated to models already developed to tackle the identification of potential hotspots and post- EPHP monitoring.

### What are the data needs?

From a modelling perspective, additional high quality data never hurts. However, since data can be so challenging to acquire, modelling techniques need to be adjusted for the limitations of data. When paramaterization is challenging, models can highlight the specific type of data that would be particularly useful. A key challenge will be to maintain the advances in the reproducibility and reliability of TF prevalence surveys. Meanwhile, as new data elements such as serology are incorporated into models, it will be important to understand the measurement process so that relevant observation bias can be incorporated.

## Data availability

No data are associated with this article.
