# Supplementary material for: Insights from mathematical modelling and quantitative analysis on the proposed 2030 goals for trachoma
Source: Gates Open Res. 2021 Mar 11;3:1721. Originally published 2019 Dec 3. [Version 2] doi: 10.12688/gatesopenres.13086.2 (PMC8111938; doi:10.12688/gatesopenres.13086.2)
Supplement: Peer review history

# Peer review: Reviewer response for version 2

**DOI:** 10.21956/gatesopenres.14449.r30503

**Published:** 2021-05-10

**Authors:** Isobel M. Blake

**Affiliations:** 1 Department of Infectious Disease Epidemiology, Imperial College London, London, UK

I have reviewed this new version and the authors have responded to my original comments sufficiently. I would like to thank the authors for taking the time to do this and for their response. I am happy for the status to be amended to ‘Approved’.

Does the article adequately reference differing views and opinions?

Yes

Are all factual statements correct, and are statements and arguments made adequately supported by citations?

Partly

Is the Open Letter written in accessible language?

Yes

Where applicable, are recommendations and next steps explained clearly for others to follow?

Partly

Is the rationale for the Open Letter provided in sufficient detail?

Yes

Reviewer Expertise:

NA

I confirm that I have read this submission and believe that I have an appropriate level of expertise to confirm that it is of an acceptable scientific standard.

# Peer review: Reviewer response for version 2

**DOI:** 10.21956/gatesopenres.14449.r30502

**Published:** 2021-04-06

**Authors:** Lisa A. Rotondo, Rebecca Flueckiger

**Affiliations:** 1 RTI International, Washington, DC, USA

It does appear that the authors have addressed all concerns.

Does the article adequately reference differing views and opinions?

Yes

Are all factual statements correct, and are statements and arguments made adequately supported by citations?

Partly

Is the Open Letter written in accessible language?

Partly

Where applicable, are recommendations and next steps explained clearly for others to follow?

Yes

Is the rationale for the Open Letter provided in sufficient detail?

Yes

Reviewer Expertise:

NA

We confirm that we have read this submission and believe that we have an appropriate level of expertise to confirm that it is of an acceptable scientific standard.

# Peer review: Reviewer response for version 1

**DOI:** 10.21956/gatesopenres.14228.r28446

**Published:** 2020-02-10

**Authors:** Lisa A. Rotondo, Rebecca Flueckiger

**Affiliations:** 1 RTI International, Washington, DC, USA

This review describes the application of mathematical modelling and analyses in informing approaches to reach the elimination of trachoma as a public health problem. The authors focus on the “active stage of the disease”, trachomatous inflammation-follicular (TF), and the challenges around the A, F and E components of the SAFE strategy and measuring prevalence of TF in low endemic settings.

 Throughout the paper acronyms are not consistently defined when they are first used and the terms district/region/evaluation unit are used interchangeably. I suggest the authors edit the review for consistency.

**Background:**

- The authors describe the natural pathway of progression from TF to TT. This is a key piece of information for the reader to understand and the current language in this section is unclear. I suggest rewording the sentence in the first paragraph of the background starting “Repeated infection with the bacteria...”. I also suggest changing the citation to Mabey *et al.* (2003 ^1^).
- In the second paragraph the authors describe the elimination targets and the intervention strategy. However, background on how the targets are measured is not included. I think it would be helpful for the reader to be provided with some background on the current clinical grading practices to set the stage for the future sections where the authors discuss challenges with the current practices in the context of low endemicity.
- Also, in the second paragraph, the authors describe three goals. We’d recommend the language of the first goal be more accurate and remove the language “2 years after MDA interventions have halted”.  It is “at least 2 years” and this is not typically included in the standard WHO language. 
- In the second paragraph the authors state, “MDA is provided to all districts where TF is >5%”. Is this accurate? I think it is probably more correct to say the MDA is *recommended*, as not all districts are able to conduct MDA for a variety of reasons. Also, the section describes MDA recommendations where TF is between 10-30%. What are the recommendations for areas where TF is >30%?
- In the fourth paragraph the authors state, “WHO is planning to revise the timeline, with the aim of achieving EPHP in all endemic districts by 2030”. The same idea is presented in Table 1 and in the abstract. This is not consistent with our understanding; the latest draft WHO NTD roadmap for 2030 is more nuanced and does not target “all endemic districts”. Is there a citation for the targets the authors are using?  If not, we recommend the publicly available documents from WHO on their target revision and the new roadmap: https://www.who.int/neglected_diseases/mediacentre/Intro_to_Roadmap_Narrative_v3.pdf
- It may also be useful to note that the original 2020 goals were political and aspirational, not based on data. Thanks to the efforts of ministries of health and countless donors and partners, the global campaign goals can now be based on data. 

 

**Section 2:**

- In the fourth paragraph the authors note, “To date it has been challenging to measure the true impact of F&E…”. Why is this the case? Is there a citation that can backup this statement?

**Section 3:**

- In the first paragraph I suggest citing Stelmach *et al.* (2019 ^2^).
- There is a typo in the last sentence of the first paragraph “underlying epidemiology”.

 

**Table 2:**

- In the geospatial survey design for TT section, I suggest citing “Flueckiger *et al.* (2019 ^3^).

Does the article adequately reference differing views and opinions?

Yes

Are all factual statements correct, and are statements and arguments made adequately supported by citations?

Partly

Is the Open Letter written in accessible language?

Partly

Where applicable, are recommendations and next steps explained clearly for others to follow?

Yes

Is the rationale for the Open Letter provided in sufficient detail?

Yes

Reviewer Expertise:

trachoma epidemiology

We confirm that we have read this submission and believe that we have an appropriate level of expertise to confirm that it is of an acceptable scientific standard, however we have significant reservations, as outlined above.

## References

[1] : Trachoma. *The Lancet*.2003;362(9379) : 10.1016/S0140-6736(03)13914-1 223-229 10.1016/S0140-6736(03)13914-1 12885486

[2] : The costs of monitoring trachoma elimination: Impact, surveillance, and trachomatous trichiasis (TT)-only surveys. *PLoS Negl Trop Dis*.13(9) : 10.1371/journal.pntd.0007605 e0007605 10.1371/journal.pntd.0007605 31487281PMC6728015

[3] : Understanding the spatial distribution of trichiasis and its association with trachomatous inflammation-follicular. *BMC Infect Dis*.2019;19(1) : 10.1186/s12879-019-3935-1 364 10.1186/s12879-019-3935-1 31039737PMC6492377

# Response

**Published:** 2021-02-02

**Authors:** Joaquin M. Prada

**Affiliations:** University of Surrey, UK

*We thank the reviewers for their insightful comments and suggestions. We have addressed all suggestions, as well as updated the text for additional clarify. Our response below in italics.*

 Reviewers 2:

 This review describes the application of mathematical modelling and analyses in informing approaches to reach the elimination of trachoma as a public health problem. The authors focus on the “active stage of the disease”, trachomatous inflammation-follicular (TF), and the challenges around the A, F and E components of the SAFE strategy and measuring prevalence of TF in low endemic settings.

 Throughout the paper acronyms are not consistently defined when they are first used and the terms district/region/evaluation unit are used interchangeably. I suggest the authors edit the review for consistency.

*We have updated throughout to “health district” for consistency.*

**Background:**

- The authors describe the natural pathway of progression from TF to TT. This is a key piece of information for the reader to understand and the current language in this section is unclear. I suggest rewording the sentence in the first paragraph of the background starting “Repeated infection with the bacteria...”. I also suggest changing the citation to Mabey *et al.* (2003 1).

*We have updated the text to clarify the pathway of progression, as suggested, and added the reference.*

- In the second paragraph the authors describe the elimination targets and the intervention strategy. However, background on how the targets are measured is not included. I think it would be helpful for the reader to be provided with some background on the current clinical grading practices to set the stage for the future sections where the authors discuss challenges with the current practices in the context of low endemicity.

*Added: “Currently, the prevalence of clinically active trachoma is assessed by trained graders’ clinical examination. In the context of low endemicity, other methods are being considered, including photographic and laboratory assessment.”*

- Also, in the second paragraph, the authors describe three goals. We’d recommend the language of the first goal be more accurate and remove the language “2 years after MDA interventions have halted”.  It is “at least 2 years” and this is not typically included in the standard WHO language. 

*We have clarified the text and aligned it with WHO language.*

  

- In the second paragraph the authors state, “MDA is provided to all districts where TF is >5%”. Is this accurate? I think it is probably more correct to say the MDA is *recommended*, as not all districts are able to conduct MDA for a variety of reasons. Also, the section describes MDA recommendations where TF is between 10-30%. What are the recommendations for areas where TF is >30%?

*We have updated and clarified the text.*

  

- In the fourth paragraph the authors state, “WHO is planning to revise the timeline, with the aim of achieving EPHP in all endemic districts by 2030”. The same idea is presented in Table 1 and in the abstract. This is not consistent with our understanding; the latest draft WHO NTD roadmap for 2030 is more nuanced and does not target “all endemic districts”. Is there a citation for the targets the authors are using?  If not, we recommend the publicly available documents from WHO on their target revision and the new roadmap: https://www.who.int/neglected_diseases/mediacentre/Intro_to_Roadmap_Narrative_v3.pdf

*We have clarified that the aim is validation of EPHP throughout the text.*

  

- It may also be useful to note that the original 2020 goals were political and aspirational, not based on data. Thanks to the efforts of ministries of health and countless donors and partners, the global campaign goals can now be based on data. 

*We have added this in the fourth paragraph.*

**Section 2:**

- In the fourth paragraph the authors note, “To date it has been challenging to measure the true impact of F&E…”. Why is this the case? Is there a citation that can backup this statement?

*We have clarified that* *field studies that have assessed F&E have been unable to find a significant effect* *and added* *three* *references (Lockwood et al, 2014; Stoller et al. 2011, Ejere et al. 2015).*

**Section 3:**

- In the first paragraph I suggest citing Stelmach *et al.* (2019 2).

*Added the reference.*

  

- There is a typo in the last sentence of the first paragraph “underlying epidemiology”.

*Corrected.*

**Table 2:**

- In the geospatial survey design for TT section, I suggest citing “Flueckiger *et al.* (2019 3).

*Reference added*

# Peer review: Reviewer response for version 1

**DOI:** 10.21956/gatesopenres.14228.r28315

**Published:** 2020-01-09

**Authors:** Isobel M. Blake

**Affiliations:** 1 Department of Infectious Disease Epidemiology, Imperial College London, London, UK

This is a well written review describing the insights mathematical and statistical analyses have provided on the 2030 goals of trachoma. Recent research is adequately cited, and the article outlines future work that needs to be addressed to help reach these goals.

 One point which is unclear to the reader is the geographic scale of reaching the 2030 target. The abstract states that 2030 is a realistic timeline for EPHP in “most” trachoma endemic areas. It is unclear to the reader the geographical scope of how many ‘areas’ there are and what does “most” mean? Are there any countries where the prevalence of TF is unknown? Have there been any quantitative analyses showing the expected time delays on mapping, and initiating MDA to know whether EPHP is achievable if there are countries that are yet to initiate control?

 The text outlines the limitations of modelling, of which a large component is the need for more empirical data to inform the analyses to hence support control guidelines. Perhaps a summary box outlining the key data needs would help strengthen this message.

 There are also some minor clarifications that could be made to the text:

1. The text uses different spatial definitions: district, area, evaluation unit and it is not clear to the reader whether these refer to the same spatial unit or not.
2. Please provide a reference for which countries have achieved EPHP or state what these countries are. On page 4 last paragraph, it says no re-emergence has been detected in districts that have eliminated trachoma. Were any of these settings hyperendemic when SAFE started? Are they representative of current high prevalence settings where F&E might be low?
3. The absence of *C. trachomatis* in children with TF seems an important phenomenon to understand better for the 2030 goals. Is there data indicating the duration of TF clearance in the absence of *C. trachomatis* absence? Is the delay a few weeks or substantially longer? As there are other causes of TF as the authors state, will there be scenarios where the trachoma control will need to continue despite the absence of *C. trachomatis* or might the guidelines be revised to aim to eliminate *C. trachomatis*?
4. Page 4 last paragraph, it says the short duration of infection is a problem for PCR. Could you expand on this? Do you mean there is a shorter time window to detect infection compared to the time window to observe TF?
5. MDA coverage is touched on in one section at the top of page 5 but have there been any quantitative analyses to investigate variability in coverage across different geographies? I think there needs to be a reference to support the statement that coverage is reported to be high.

Does the article adequately reference differing views and opinions?

Yes

Are all factual statements correct, and are statements and arguments made adequately supported by citations?

Partly

Is the Open Letter written in accessible language?

Yes

Where applicable, are recommendations and next steps explained clearly for others to follow?

Partly

Is the rationale for the Open Letter provided in sufficient detail?

Yes

Reviewer Expertise:

Infectious Disease Epidemiology

I confirm that I have read this submission and believe that I have an appropriate level of expertise to confirm that it is of an acceptable scientific standard, however I have significant reservations, as outlined above.

# Response

**Published:** 2021-01-29

**Authors:** Joaquin M. Prada

**Affiliations:** University of Surrey, UK

*We thank both reviewers for their insightful comments and feedback. We have clarified and addressed all their comments, as well as updated the text to add additional clarity where needed. We have added our response in italics below.*

 Reviewer 1:

 This is a well written review describing the insights mathematical and statistical analyses have provided on the 2030 goals of trachoma. Recent research is adequately cited, and the article outlines future work that needs to be addressed to help reach these goals.

 One point which is unclear to the reader is the geographic scale of reaching the 2030 target. The abstract states that 2030 is a realistic timeline for EPHP in “most” trachoma endemic areas. It is unclear to the reader the geographical scope of how many ‘areas’ there are and what does “most” mean? Are there any countries where the prevalence of TF is unknown? Have there been any quantitative analyses showing the expected time delays on mapping, and initiating MDA to know whether EPHP is achievable if there are countries that are yet to initiate control?

*We have clarified in the abstract that elimination as a public health problem is for all endemic health districts (in all endemic countries). Current goal set by WHO is to validate 64 countries by 2030. We have also updated the text throughout to clarify the spatial units we refer to are health districts (which are the practical implementation units of the interventions).*

 The text outlines the limitations of modelling, of which a large component is the need for more empirical data to inform the analyses to hence support control guidelines. Perhaps a summary box outlining the key data needs would help strengthen this message.

*We apologize if we came across as requesting more and more data! That’s the perennial excuse of model* *l* *ers. More data is always nice. However, since data can be so challenging to acquire, we need to adjust our modelling techniques for the limitations of data. Such models could also highlight which data would be particularly useful. We have added a section at the end of the manuscript to this effect.*

 There are also some minor clarifications that could be made to the text:

- The text uses different spatial definitions: district, area, evaluation unit and it is not clear to the reader whether these refer to the same spatial unit or not.

*We have now for consistency used the term health district throughout (as mentioned above, these are the implementation units) or alternatively countries.*

  

- Please provide a reference for which countries have achieved EPHP or state what these countries are. On page 4 last paragraph, it says no re-emergence has been detected in districts that have eliminated trachoma. Were any of these settings hyperendemic when SAFE started? Are they representative of current high prevalence settings where F&E might be low?

*We have reworded this to clarify that there is* *no evidence that re-emergence can’t be explained by misclassification error* *(Godwin et al 2020)* *.* *We* *have also* *added a web citati* *on for the number of countries achieving EPHP.*

- The absence of *C. trachomatis* in children with TF seems an important phenomenon to understand better for the 2030 goals. Is there data indicating the duration of TF clearance in the absence of *C. trachomatis* absence? Is the delay a few weeks or substantially longer? As there are other causes of TF as the authors state, will there be scenarios where the trachoma control will need to continue despite the absence of *C. trachomatis* or might the guidelines be revised to aim to eliminate *C. trachomatis*?

*We have clarified in the text that at the community level, TF resolves slowly (Keenan et al, 2011).* *Moreover* *, the non-linear relationship between viral load and TF makes understanding the relationship between PCR and TF challenging.*

- Page 4 last paragraph, it says the short duration of infection is a problem for PCR. Could you expand on this? Do you mean there is a shorter time window to detect infection compared to the time window to observe TF?

*We have clarified this in the text, indeed, the time window for detect positive individuals by PCR is smaller. Moreover, modelling work has shown that including PCR data does not significantly improve forecasts of TF (Liu et al. 2015).*

- MDA coverage is touched on in one section at the top of page 5 but have there been any quantitative analyses to investigate variability in coverage across different geographies? I think there needs to be a reference to support the statement that coverage is reported to be high.

*We have added a reference for this statement (Astale et al. 2018).*
